# QRS Fragmentation Patterns Representing Myocardial Scar Need to Be Separated from Benign Normal Variants: Hypotheses and Proposal for Morphology based Classification

**DOI:** 10.3389/fphys.2016.00653

**Published:** 2016-12-26

**Authors:** M. Anette E. Haukilahti, Antti Eranti, Tuomas Kenttä, Heikki V. Huikuri

**Affiliations:** ^1^Research Unit of Internal Medicine, Medical Research Center, University Hospital of OuluOulu, Finland; ^2^Department of Internal Medicine, Päijät-Häme Central HospitalLahti, Finland

**Keywords:** fragmented QRS, electrocardiography, prognosis, diagnosis, cardiac arrhythmias, cardiac diseases, myocardial scar, sudden cardiac death

## Abstract

The presence of a fragmented QRS complex (fQRS) in two contiguous leads of a standard 12-lead electrocardiogram (ECG) has been shown to be an indicator of myocardial scar in multiple different populations of cardiac patients. QRS fragmentation is also a predictor of adverse prognosis in acute myocardial infarction, coronary artery disease, and ischemic cardiomyopathy and a prognostic tool in structural heart diseases. An increased risk of sudden cardiac death associated with fQRS has been documented in patients with ischemic cardiomyopathy and hypertrophic cardiomyopathy. However, fQRS is also frequently observed in apparently healthy subjects. Thus, a more detailed classification of different QRS fragmentations is needed to identify the pathological fragmentation patterns and refine the role of fQRS as a risk marker of adverse cardiac events and sudden cardiac death. In most studies fQRS has been defined by the presence of an additional R wave (R′), or notching in the nadir of the S wave, or the presence of >1 R′ in two contiguous leads corresponding to a major coronary territory. However, this approach does not discriminate between minor and major fragmentations and the location of the fQRS is also neglected. In addition to this, the method is susceptible to large interobserver variability. We suppose that some fQRS subtypes result from conduction delays in the His-Purkinje system, which is a benign finding and thus can weaken the prognostic values of fQRS. The classification of fQRSs to subtypes with unambiguous definitions is needed to overcome the interobserver variability related issues and to separate fQRSs caused by myocardial scarring from benign normal variants. In this paper, we review the anatomic correlates of fQRS and the current knowledge of prognostic significance of fQRS. We also propose a detailed fQRS classification for research purposes which can later be simplified after the truly pathological morphologies have been identified. The research material of our study consist of 15,245 ECGs from the random general population and approximately six thousands (*n* = 6,241) ECGs from subjects with a known cardiac disease.

## Backgrounds

### The formation of the QRS fragmentation patterns

The occurrence of fQRS was first noticed in 1973 by Boineau and Cox (1973) when experimenting on acute ischemia on canine hearts. The mechanism of formation of the fQRS can be explained by significant scarring and necrosis of the myocardium (Zipes and Das, 2009; Fares et al., 2013; Jain et al., 2014). In 2006 Das et al. proved by myocardial single photon emission tomography (SPECT) in a population of patients referred to a nuclear stress test that the presence of fQRS correlates to a regional myocardial scar and the sensitivity of fQRS to detect myocardial scar is significantly greater (85.6%) than the sensitivity the Q wave has (36.3%) (Das et al., 2006). The location of fQRS in the ECG has also generally correlated well with the location of myocardial scar (Zipes and Das, 2009). Lorgis et al. (2014) showed by cardiac magnetic resonance imaging (cMRI) that among patients with ST elevation myocardial infarction (STEMI) fQRS is a marker of infarct size and acute ventricular remodeling. In their studies, the presence of fQRS was associated with a larger infarct size and peri-infarct zone, myocardial perfusion abnormalities, increased heart volumes on the left side of the heart and lower left ventricular ejection fraction (LVEF). In a population of stable coronary artery disease (CAD) patients with a chronic total occlusion the prevalence of fQRS has been shown to be greater among those with poor collateral circulation or myocardial scar (Bonakdar et al., 2016). Scar tissue and ischemic regions in the myocardium cause nonhomogenous activation due to regional conduction slowing or block that can lead to the ECG findings considered as fQRS (Das et al., 2007).

In patients with hypertrophic cardiomyopathy (HCM) the number of leads with fQRS morphology and also the location of fragmentation has been shown to correlate with the amount and location of areas showing late gadolinium enhancement in cMRI indicating myocardial scarring (Konno et al., 2015). HCM is not only characterized with cellular hypertrophy but also tissue heterogeneity such as myofiber disarray or interstitial, diffuse fibrosis. According to one study fQRS in lateral leads showed the highest accuracy for myocardial fibrosis. Debonnaire et al. (2015). In addition, it has been noticed (Adar et al., 2015) that radiotherapy for breast cancer induces formation of fQRS on the ECG with an independent contribution of cardiac radiation dose. One of the well-known side effects of the radiotherapy is myocardial fibrosis. In other words, fQRS changes can also originate from more diffuse scarring processes than myocardial infarction (MI).

In cardiac sarcoidosis there is formation of myocardial granuloma and scarring, and an increased prevalence of fQRS has been observed in patients suffering the disease (Homsi et al., 2009). Thus, fQRS may represent a local conduction slowing in the ventricular myocardium caused by either scar, fibrosis, inflammation, or ischemia. These actions shift the QRS vectors as a distribution of the normal electrical activation of the ventricles which may produce the different RSR′-patterns (Das et al., 2007; Sha et al., 2011). However, some of the pathologic processes that result in fQRS are reversible. Among the scarred tissue there can be regions of viable myocardial tissue (Zipes and Das, 2009; Fares et al., 2013) and ischemia decelerates the conduction velocity in the peri-infarct zone (Mann et al., 2015). It has been shown that fQRS may diminish from the ECG during a cardiac rehabilitation program after a STEMI and patients with hypertension (Bulut et al., 2015). Resolution of fQRS has also been detected among patients responding favorable to cardiac resynchronization therapy (Celikyurt et al., 2015). This can be explained by improved electrical stability in the myocardium and it is also related to increase in survival and decrease in major cardiac events.

### Prognostic significance of the fragmented QRS complex in cardiomyopathies and structural heart diseases

fQRS is frequently observed in subjects with ischemic and non-ischemic cardiomyopathy and in subjects with other structural cardiac diseases. For example, fQRS can be exploited in identification of the arrhythmogenic right ventricular dysplasia-cardiomyopathy and detection of left ventricle aneurysms (Fares et al., 2013). When occurring ≥3 major coronary artery territories fQRS is independently associated with ventricular tachyarrhythmias (VTA) and sudden cardiac death (SCD) in patients with HCM (Debonnaire et al., 2015). In this study 75% of the HCM patients displayed fQRS at least one coronary artery territory, most commonly in the inferior territory. It seems that fQRS in HCM patients could reflect the vulnerable structural substrate that is needed for incidence of re-entry VTA (Debonnaire et al., 2015).

Especially when occurring in the lateral territory fQRS has been noticed to increase not only the non-fatal cardiac events and long-term cardiovascular events but also mortality in patients with CAD (Jain et al., 2014; Gong and Li, 2016; Güngör et al., 2016). It is possible that fQRS could detect some subclinical regional left ventricle dysfunction in patients with CAD (Yan et al., 2012). In patients with acute coronary syndrome fQRS has shown to be an independent predictor for mortality (Das et al., 2009). When studying patients with non-ST elevation myocardial infarction (NSTEMI) (Das et al., 2009) fQRS was detected on the ECG of 51% of MI patients and only 3.7% of the patients with unstable angina who served as a control group. fQRS can also be associated with low LVEF. (Fares et al., 2013; Lorgis et al., 2014; Gong and Li, 2016; Güngör et al., 2016; Ma et al., 2016) and multi-vessel CAD in patients with STEMI (Ma et al., 2016) and NSTEMI (Güngör et al., 2016) which may explain the association with adverse prognosis. In silent MIs, which are problematic to discover in diabetics, women with unusual chest pain and in senile dementia, fQRS can be the only evidence of the ongoing event (Fares et al., 2013).

### QRS fragmentation in the prediction of arrhythmias

fQRS can be used as an ECG tool in prediction of arrhythmias. According to the previous studies (Das and El Masry, 2010; Das et al., 2010; Jain et al., 2014) of fragmented QRS as a risk marker in cardiovascular diseases, fQRS predicts ventricular arrhythmia in patients with CAD. Some studies have identified fQRS as an independent predictor of SCDs (Das et al., 2010; Terho et al., 2014). In idiopathic dilated cardiomyopathy fQRS has shown to predict both ventricular tachycardias and all-cause mortality (Sha et al., 2011). Also a greater risk of developing Torsades des pointes has been shown to be associated with fQRS in patients with acquired long QT syndrome (Fares et al., 2013). In addition, fQRS predicts both arrhythmic events and mortality in patients with implantable cardioverter defibrillator (ICD) (Jain et al., 2014).

Also the relationship between fQRS and monomorphic ventricular tachycardia has been observed. In patients with ventricular tachycardias fragmented QRS complexes have longer durations which may be due to the correspondence with the fQRS site and the regions where re-entry occurs. This could be one explanation for the underlying mechanisms of the ventricular tachycardia (Fares et al., 2013). A higher amount of fragmented QRS complexes are also seen in Brugada syndrome patients with SCN5A mutation compared to those lacking the mutation (Fares et al., 2013). Therefore, Morita et al. (2008) suggested that fQRS may be an useful ECG tool for identifying Brugada syndrome. However, another review (Jain et al., 2014) claims fQRS reflecting poor prognosis in patients with Brugada syndrome and arrhythmogenic right ventricular cardiomyopathy. Now, it is currently studied if fQRS could be used as a risk stratification tool when evaluating the need for invasive therapy or device therapy. Especially left ventricle aneurysms are prone to ventricular arrhythmias and treatable with ablation. fQRS can be taken advantage of identifying the potential areas for the treatment. fQRS is also a non-invasive method when making the decision for the ICD implantation (Fares et al., 2013).

### Reasons for the morphology based QRS fragmentation classification

Most research groups who have studied the link between the SCD and the fQRS have used the fQRS criteria proposed by Das et al. (2006). Das et al. defined fQRS as an additional R wave (R′) or notched S wave. According to the fQRS criteria proposed by Das et al. there can be one or more additional R waves in the fragmented QRS complex (R′ > 1). The R′ can be spiked or slurred. The conditions for fQRS in the criteria of Das et al. are that fQRS have to exist in two or more contiguous leads and the width of QRS complex must be <120 ms. However, there are no conditions for the amplitude of the fQRS. Also the location of the fQRS in the QRS complex has been neglected. Broader QRS complexes have their own fQRS criteria, also developed by Das et al. (2006).

We aim at developing a more specific and unambiguous fQRS criteria. With the current fQRS criteria the repeatability of the measuring between different subjects and clinics is quite weak (Malik, 2013). With the current criteria the utility of fQRS as a risk marker alternates considering the population studied and the incidence of ventricular disease in it (MacAlpin, 2010). When we take a look at some of the studies considering fQRS as a risk factor of SCD with study populations similar to the original study of Das et al. (2006) the findings are contradictory. Wang et al. (2010) studied ECG and nuclear perfusion images of 460 patients with a known CAD. In their study the sensitivity of fQRS was worse (1.7%) than the sensitivity of Q wave (31.7%) for identifying myocardial scar. Their recent findings (Wang et al., 2014) are also similar: fQRS cannot be supported as a reliable predictor for SPECT myocardial scar, major adverse cardiac events or all-cause mortality based on a study with along follow-up. In addition to this, another study (Ahn et al., 2013) claims that neither fQRS or Q waves are valuable prognostic tools when diagnosing the transmural irreversible myocardial injury in the case of acute myocardial infarction.

Thus, the diagnostic accuracy of fQRS in detecting myocardial scar and prognostic significance of fQRS in different populations have varied substantially. In addition to differences between populations studied, one conceivable reason for the controversies could be the measuring differences between the clinics (Malik, 2013). However, we believe that the most important reason for the variation is that some fQRS morphologies are benign normal variants whereas other morphologies represent myocardial scarring. This is supported by the high prevalence of fQRS in apparently healthy populations. Terho et al. (2014) observed inferior fQRS in 15.6%, anterior in 2.9% and lateral in 0.5% of the subjects without a known cardiac disease in a general population sample of over 10,000 middle-aged subjects. Respectively, in patients with a known cardiac disease 16.7% had inferior, 3.8% anterior and 1.8% lateral fQRS. It is possible (Terho et al., 2014) that some fQRS morphologies could be an early marker of subclinical cardiac disease in those without a known cardiac disease. Therefore, there is a need for a more detailed fQRS classification which takes into account the amplitudes of the fragmentations and the location of the fragmentations in the QRS complex. Das et al. (2006) already presented various different fQRS morphologies, but they have not been used.

**Figure 1:**
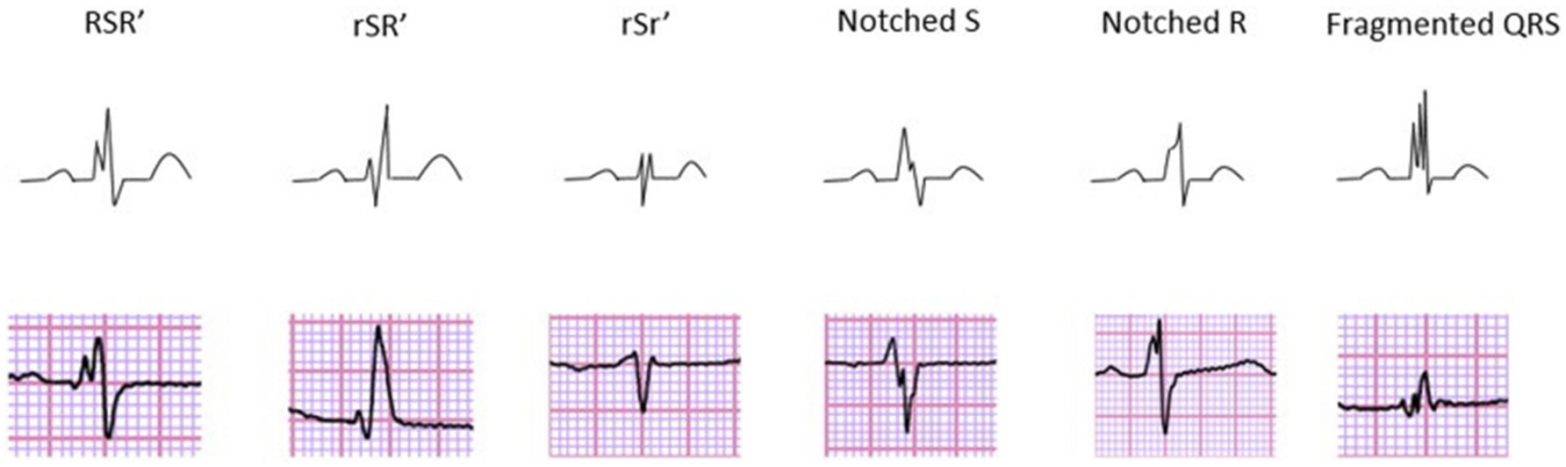
**Different morphologies of fQRS on 12-lead ECG describing the fQRS criteria proposed by Das et al**. Reprinted from the article “Prevalence and prognostic significance of fragmented QRS complex in middle-aged subjects with and without clinical or electrocardiographic evidence of cardiac disease” (Terho et al., 2014) with permission from Elsevier.

In experimenting with different fQRS classification criteria, we observed that the fQRS morphology classes presented by Das et al. (2006) (Figure 1) as especially susceptible to interobserver and even intraobserver variability. It even lacks some of the fQRS morphologies we would consider worth to familiarize with. Das et al. do not require change of slope's sign in their fQRS criteria. We found especially the inclusion of slurred changes as indicators of fQRS conflicting in ECGs recorded at paper speed of 50 mm/s. Also the separation of early repolarization patterns (ER) from fragmentation in the terminal part of the QRS complex requires attention (Macfarlane et al., 2015). Furthermore, any consensus has not been done to these fQRS criteria proposed by Das et al.

Even though, fQRS criteria proposed by Das et al. may be the mostly used criteria for fragmented QRS complex, there are also other fQRS criteria. Torigoe et al. studied fQRS considering the number of leads (Torigoe et al., 2012). The number of leads with fQRS in patients with prior myocardial infarction, especially when occurring in three or more leads, seemed to be an independent predictor for cardiac death or hospitalization for heart failure. However, the research materials in both of Das et al. and Torigoe et al. have been analyzed by on-off-criteria: leads either contain any kind of fQRS or no fQRS at all. Neither of these research groups have evaluated the prognostic values of the different fQRS morphologies.

Previously one research group has had a largely similar approach to ours. Maheshwari et al. (2013) have proposed that fQRS should be assessed in greater detail also paying attention to the morphology of the fQRS. However, because there were no standardized fQRS morphologies, Maheshwari et al. avoided publishing their morphological results obtained from an automated algorithm-based detection of fQRS. In other words any data from the prognostic values of the different fQRS morphologies have not yet been published. Maheshwari et al. classified 10 different fQRS morphologies based on earlier literature and some other variations: (A) rSr′, (B) rsR′ (notched R), (C) RsR′ with ST-elevation, (D) rSR′, E) RsR′ without ST elevation, (F) Rsr′, G) RSr′, (H) notched S, (I) RSR′, (J) f-QRS. In their study the measurements obtained with an automated algorithm correlated well with those made by two experienced cardiologists.

Even though the fQRS criteria of Maheshwari et al. (2013) are largely similar with our modified fQRS criteria also including a morphology-based classification, we think that some amendments could be beneficial. The main difference between our modified fQRS criteria to the criteria of Maheswhari et al. is that we do not observe fragmentations only time-based but also amplitude-based. We believe that fQRSs with a high amplitude deflection between the R′s would have a greater prognostic value for SCD than minor-fQRSs have. However, this approach provides a chance to study whether two minor-fQRS in contiguous leads would predict the greater risk for SCD than one major-fQRS. The time-based observation enables us to examine if the location of the fragmentation has an influence to the prognostic values.

Based on our experiences in testing different fQRS criteria, a fragmentation of the Q wave should also be documented. We also assume that it would make sense to differentiate between two or more fragmentations occurring in the different parts of the QRS complex (for example fragmented R wave and fragmented S wave) and multiple fragmentations in a single wave of the QRS complex. We have paid attention specially in naming of different fQRS morphologies to avoid as many misunderstandings as possible. Maheshwari et al. (2013) have considered always the last R wave as additional R wave. To our mind the additional R wave, however, can occur before the original R wave. This may not always produce the largest amplitude change but illustrates the myocardial conduction delay better. However, they examine fQRSs with and without Q wave and S wave as we are going to do. They also consider fragmentation present in wide QRS complexes (>120 ms) which is a great extra. We have thought to analyze >120 ms fragmented QRS complexes in the later studies and contemplate a detailed criteria for them, too.

## The introduction of the modified fQRS criteria

To clarify present fQRS criteria we have developed modified fQRS criteria which we believe to be well adaptable to research use, especially concerning searching the associations from the broad cohort studies. In the modified fQRS criteria we require occurring of a change in the sign of the slope that the ECG alteration could be considered as fragmentation. Also fragmentation in the downslope of the R wave should occur in the upper 50% of the R wave to be considered as fQRS instead of ER. In the consensus paper of early repolarization by Macfarlane published in 2015 (Macfarlane et al., 2015), ER has been defined as a positive ECG change at the end of the QRS complex. ER can be slurred or notched and it should occur on the final 50% of the downslope of an R wave. The fragmentation can also occur in the ascending part of the R wave, at the R peak or in any part of the S wave or Q wave.

**Figure 2:**
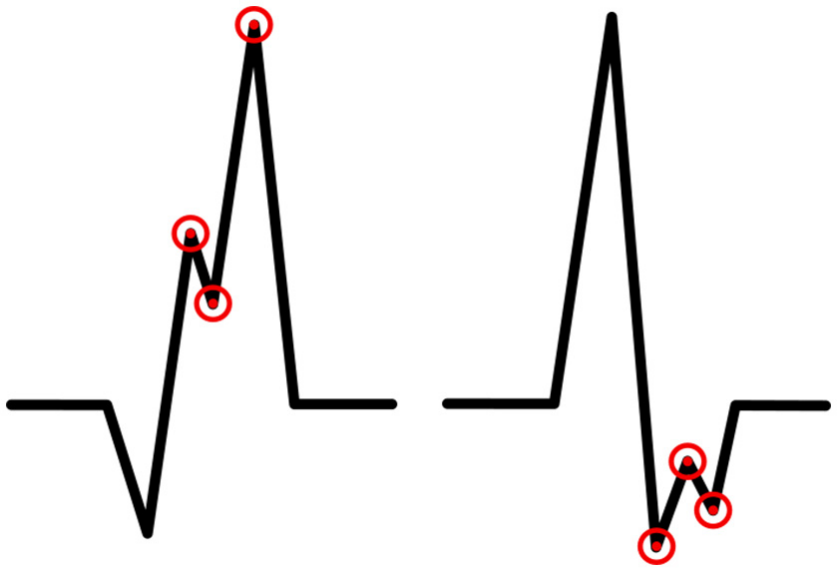
**An example of the measurement of amplitude changes and location of the fragmentations in QRS complex**. One point is tagged to the fQRS notch, the second to the fQRS peak and the third to the peak of the wave in which fQRS occurs.

While doing literature searching we did not come across with the accurate definition of R′ and r′. We defined these terms to clarify our criteria for fQRS. R′ is an additional R wave which amplitude is higher than 50% of amplitude of the highest amplitude wave (by absolute value) of the QRS complex (Q wave, R wave, or S wave). Similarly, r′ is an additional R wave which amplitude is lower than 50% of the amplitude of the highest amplitude wave (by absolute value) of the QRS complex (Q wave, R wave, or S wave). In this paper R is standardizing for the main deflection and R' the lesser deflection (fragmentation) regardless of the order they present themselves. The same regulation concerns other additional waves (S′ and Q′). In the modified fQRS criteria we require that the width of the QRS complex to be <120 ms. The fQRS does not need to occur in at least two contiguous leads because we want to study, for example, if one major-fQRS could have a greater prognostic value than two contiguous minor-fQRSs. The fQRS amplitudes will be measured by using EASE ECG-analysis computer program developed to the need in question. In every fQRS there are tagged three points: first to the notch of fragmentation, second to the peak of fragmentation and the third to the peak of the wave in which fQRS occurs (Figure 2). The amplitude differences will be measured between these points.

Instead of the six-class fQRS morphology of Das et al. (2006) and the ten-class morphology of Maheshwari et al. (2013) we have developed more detailed morphology classification of five classes and 15 subgroups. We separate fQRSs into fragmentation of Q wave, R wave and S wave. In addition to this there are four different RSR-patterns. Other forms of fragmentation are considered as a separate class. We believe this morphology classification will help us to identify the most malign fQRS morphologies form those which are supposedly benign findings. The morphology classification of the modified fQRS criteria is described below.

### Fragmentation in the Q wave

**Figure 3:**
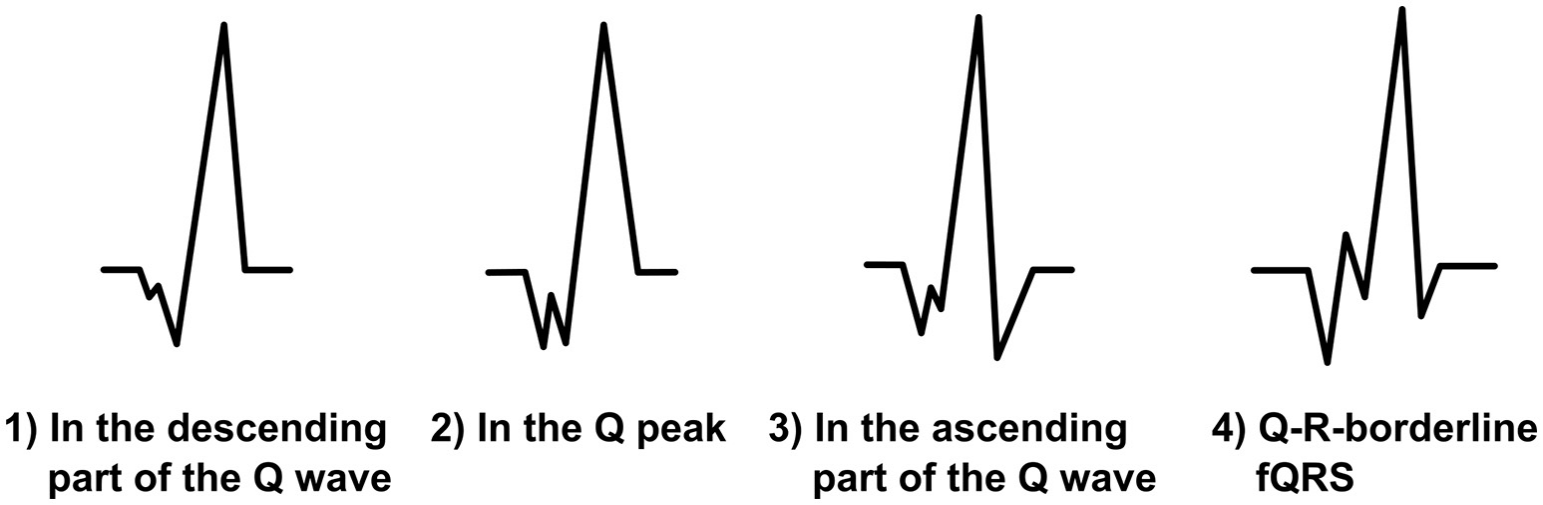
**The modified fQRS criteria: different morphologies of the fragmented Q wave and a Q-R-borderline-fQRS morphology on the right**.

When there is notching in a Q wave, the Q wave is considered as a fragmented Q wave (Figure 3). The additional Q wave (Q′) can occur in any part of descending or ascending Q wave or at the Q peak. Fragmentation must always be located under the baseline and therefore be negative. In addition, the fragmentation can occur in the borderline of the Q wave and the R wave. In this case, we call the fragmentation Q-R-borderline-fQRS (Figure 3). Now the fragmentation has both a positive and a negative change (+/−) because it is observed on the both sides of the baseline. When measuring fQRS amplitude, the third point will be tagged to the peak of the Q wave.

### Fragmentation in the R wave

**Figure 4:**
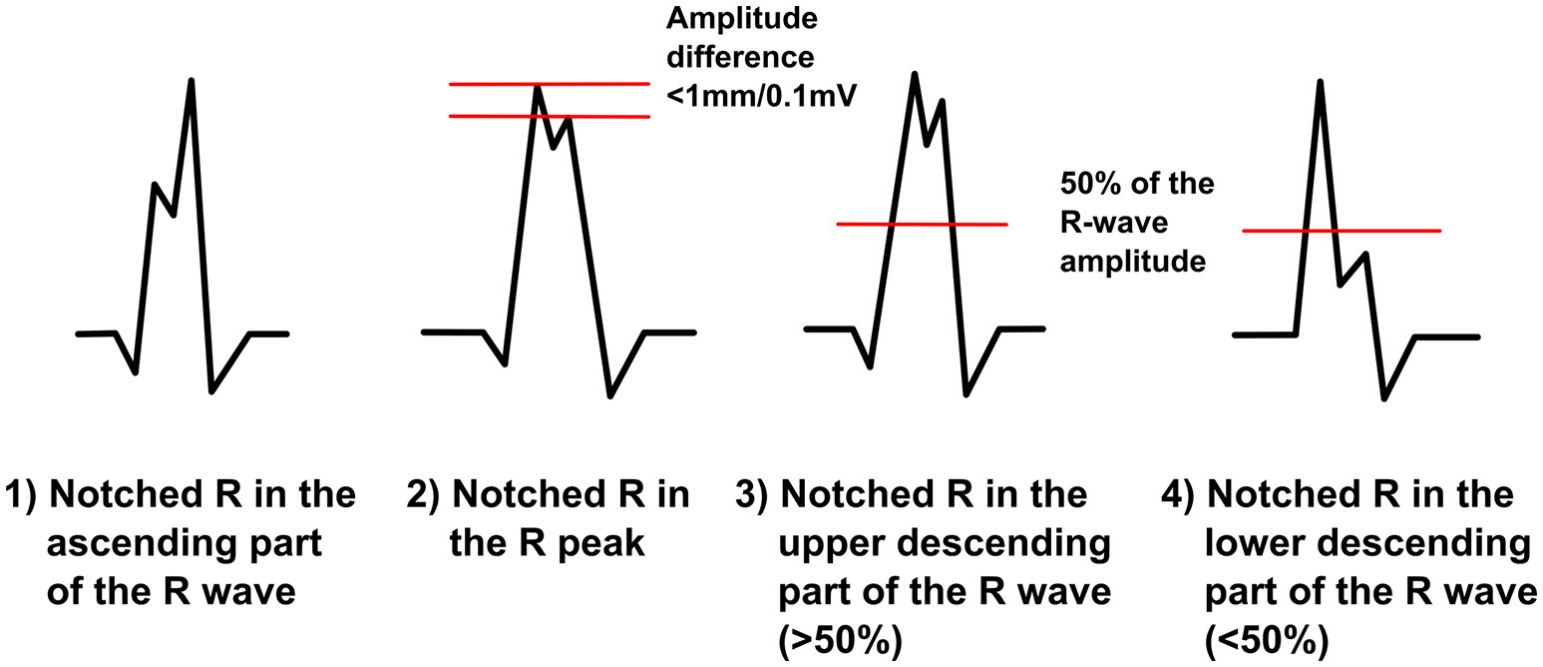
**The modified fQRS criteria: different RsR-morphologies**.

This morphology class includes different RsR-morphologies (Figure 4). We call it by common noun of “notched R.” Now fragmentation is always positive (does not go to the negative side of the baseline) and can occur in any part of ascending R wave (R′sR or r′sR), in the R peak (RsR′ or Rsr′) or in the upper part (>50%) of descending R wave (RsR′ or Rsr′). When occurring at the R peak the amplitude difference between the peak of the additional R wave and the peak of the original R wave cannot be over 1,0 mm/0,1 mV. In other words we separate notched R into the three subgroups based on the location in the time domain. In our research we will also consider Rsr′ in the lower part of downsloping R wave (in the absence of S-wave, (<50% of R amplitude) as one additional subgroup, as Das et al. did, but in the reality we perceive it more as an ER. When measuring fQRS amplitude, the third point will be tagged to the peak of the R wave.

### Fragmentation in the S wave

**Figure 5:**
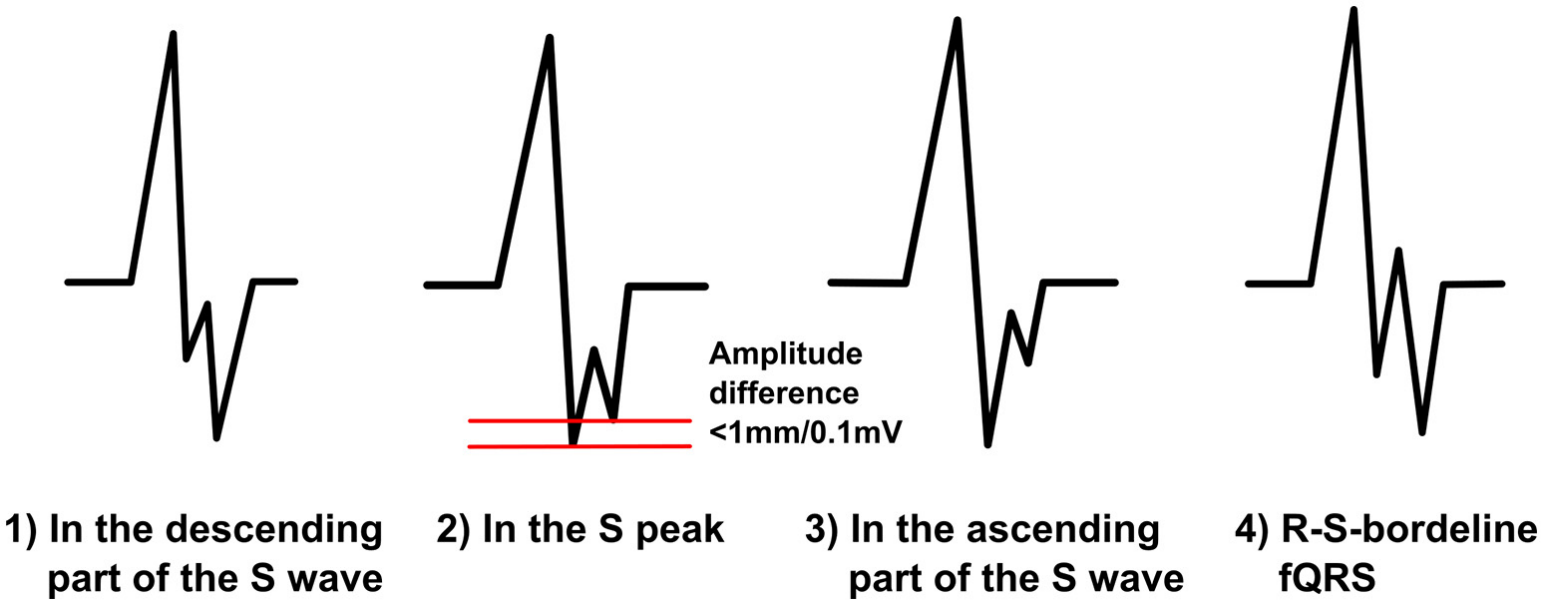
**The modified fQRS criteria: different morphologies of the notched S on the left and an example of the R-S-borderline-fQRS on the right**.

We divide the notched S-morphology of Das et al. into two separate classes: notched S and R-S-borderline-fQRS (Figure 5). We believe this will help us to compare the prognostic values of different fQRS morphologies better. When the fragmentation occurs in the descending S wave, at the S peak or in the ascending part of S wave, we call it notched S. Notched S is always a negative fQRS. We divide this class into three subgroups mentioned above considering the location of the fQRS in the time domain. When occurring at the S peak, the amplitude difference between the peak of the additional S wave and the peak of the original S wave can be, on top, 1,0 mm/0,1 mV. R-S-borderline-fQRS occurs in the borderline of the R wave and the S wave. It goes to the both sides of the baseline so that the fragmentation gets both positive and negative values. When observed from the beginning of the QRS complex the first S wave must have smaller amplitude change than the second S wave. Also the first R wave must be >50% of the amplitude of the fragmentation's R wave. These additions will help us to separate R-S-borderline morphology from the different RSR-patterns with an additional S wave. When measuring fQRS amplitude, the third point will be tagged to the peak of the S wave.

### Different RSR-patterns

In addition to fragmentations of the different waves of QRS complex described above, to our mind, there can be four different RSR-patterns separated. We will analyze the effect of a Q wave and an additional S wave (RSRS-patterns) considering these fQRS morphologies.

#### RSR′

**Figure 6:**
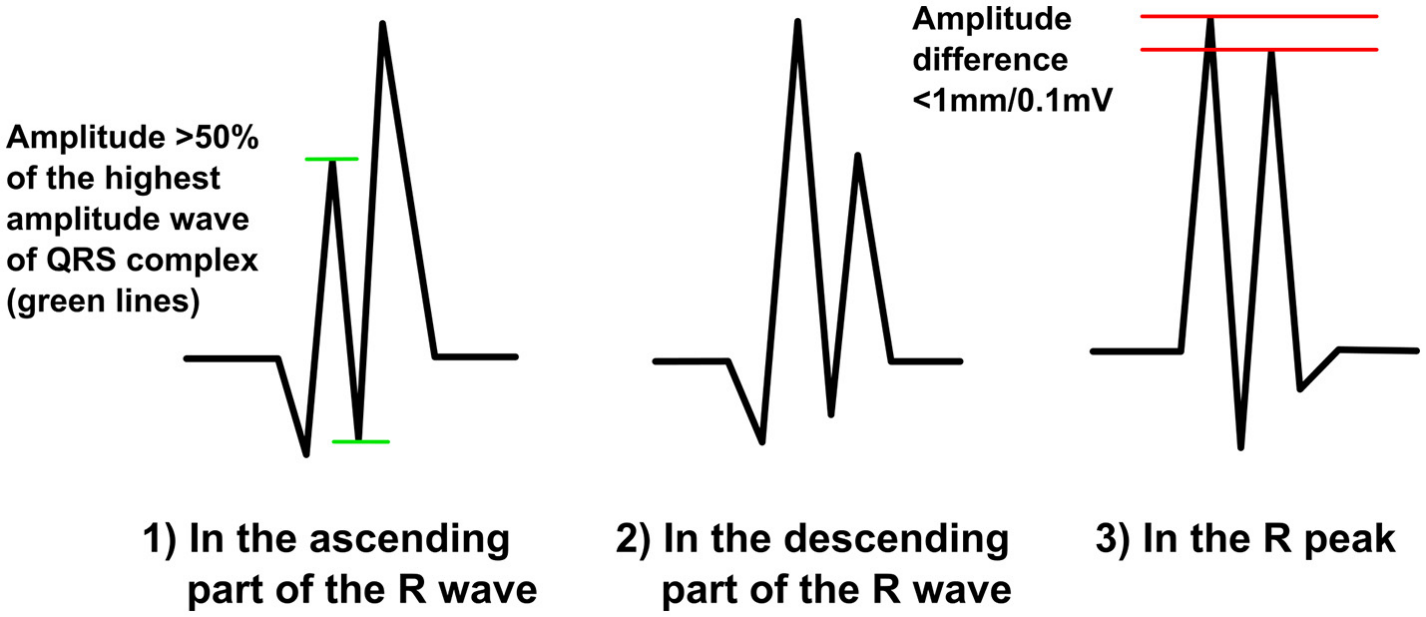
**The modified fQRS criteria: different RSR-morphologies**.

In this RSR pattern, the S wave goes below the baseline, thus separating it from the notched R-morphology, where the S′ wave remains entirely positive. The RSR pattern must fulfill the criteria for R′. An additional R wave can occur in any part of ascending R wave (R′SR), in the R peak (RSR) or in the upper part (>50%) of the descending R wave (RSR′) (Figure 6). When occuring at the R peak the amplitude difference between the peak of the additional R wave and the peak of the original R wave cannot be over 1,0 mm/0,1 mV.

If the additional R wave occurs in the ascending part of the R wave with a Q wave must the amplitude of R′ be observed precisely. If the amplitude of the R′ is smaller than 50% of the highest amplitude wave the fQRS is considered as a Q-R-borderline-fQRS instead of RSR-morphology. Again, if the additional R wave occurs in the lower part of the downsloping R wave (<50%) and the S wave between the two R waves goes under the baseline the fQRS morphology can be considered as a RSr′. The separation between these morphologies is also based on the amplitude of the additional R wave. When measuring fQRS amplitude, measurement points are tagged to the both peaks of R waves and to the S peak.

#### RSr′

**Figure 7:**
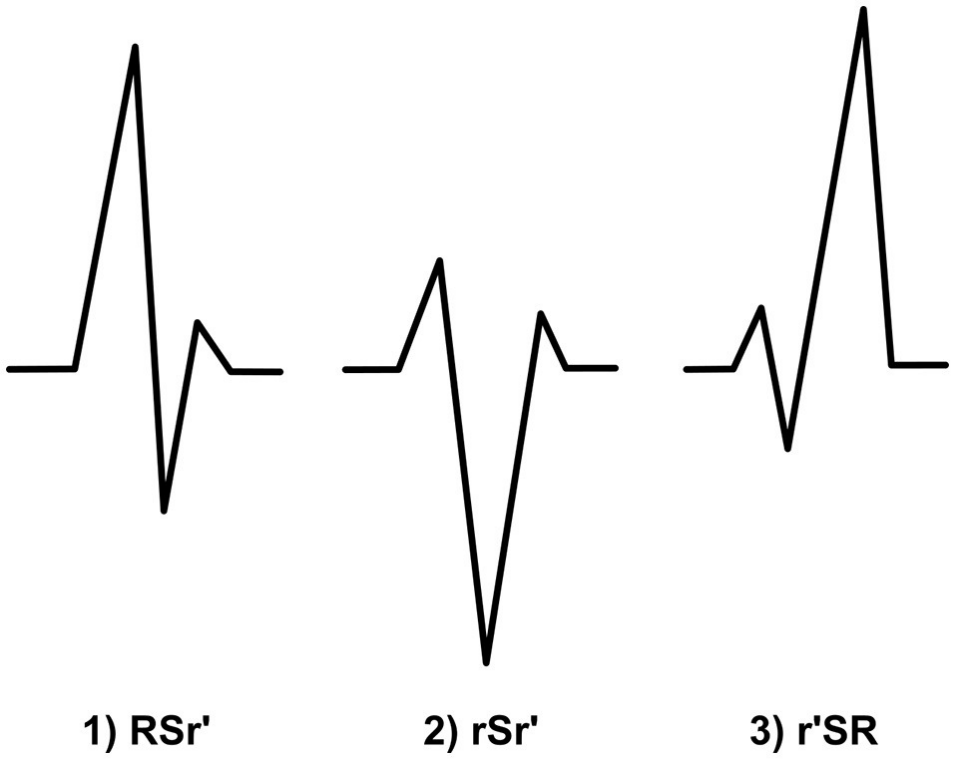
**The morphologies of the modified fQRS criteria from left to right: (1) RSr′ (2) rSr′ (3) r′SR**.

When a small r′ occurs after a large R wave and a considerable S wave (gets negative values), we categorize the fQRS to the RSr′-morphology (Figure 7). Additional r′ must fulfill the criteria of r′. Also the original R wave must be over 50% of the amplitude of the of the highest amplitude wave of the QRS complex. Q wave can be present. We expect this morphology to be a benign finding. When measuring amplitude, measurement points are tagged to the peak of the r′, the S peak and the baseline after the QRS complex or to the peak of the following S′ wave.

#### rSr′

In this fQRS morphology S wave is the highest amplitude wave. The both amplitude of r wave and the amplitude of the additional r′ must be smaller than the 50% of the amplitude of S wave (Figure 7). Thus, criteria for r′ must be fulfilled. Q wave can be present. As the previous morphology, also this fQRS morphology is supposed to lack prognostic value. When measuring amplitude, measurement points are tagged similarly to RSr′-morphology.

#### r′SR

r′SR or mini-r-fQRS, as we like to call this morphology, is a fQRS where a small r′ (the amplitude is smaller than 50% of the highest amplitude wave) occurs in the beginning of the QRS complex and is followed immediately by S wave that goes under the baseline (Figure 7). Criteria for r′ must be fulfilled. There cannot be a Q wave before the r′. If so, the fQRS is classified as a Q-R-borderline-fQRS. Our hypothesis is that this morphology dos not have much of prognostic value. When measuring amplitude, measurement points are tagged to the baseline before QRS complex, the peak of the r′ and the S peak.

### Other forms of fragmentation

**Figure 8:**
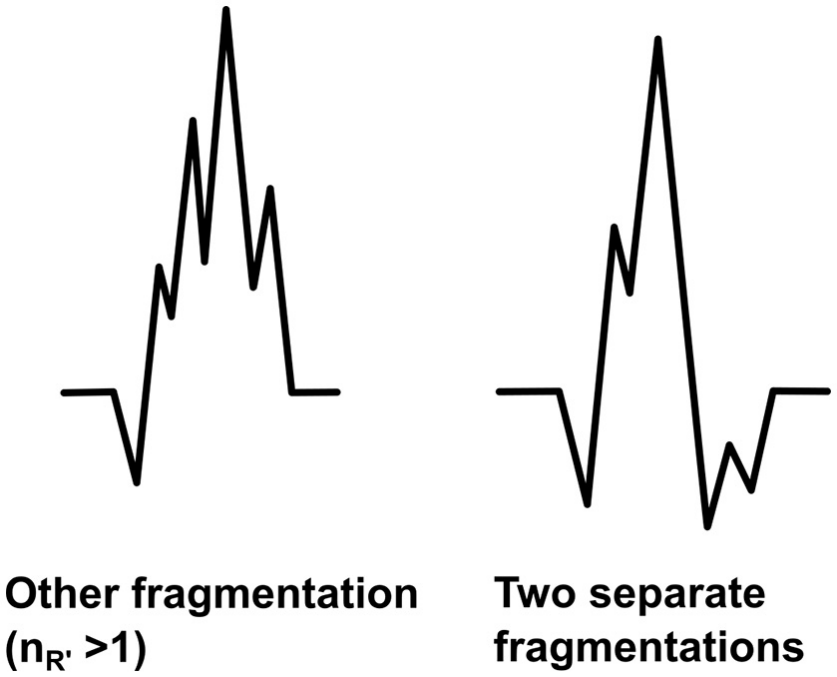
**Other forms of fQRS**. On the left the other fragmentation and on the right an example of two separate fQRS morphologies (notched R and notched S) occurring in the same QRS complex.

The fQRS will be considered as the other fragmentation if it differs from the fQRS morphologies described above. We are aimed at developing the modified fQRS criteria so that only fQRSs which have more than one additional R waves (R′ > 1) in the same wave could be considered as the other fragmentation (Figure 8). When measuring the amplitude differences two points are tagged to measure the largest amplitude difference of the fragmentation and the third point to measure the larger amplitude difference next to the largest change. However, this morphology class includes also fragmented QRS complexes which include more than one fragmentation in separate waves of the QRS complex (Figure 8). In these cases the both different fQRS morphologies will be examined separately to ensure that any potential fQRS morphology finding will not go unnoticed.

## Hypothesis

We expect that by using more detailed modified fQRS criteria it would be possible to separate fQRSs that are developed due to intramyocardial conduction delay caused by the myocardial scarring or ischemia from those due to benign conduction delay or anatomical variation in the His-Purkinje system. For example, right bundle-branch block with a typical R′ in the leads V1-V3 is not associated with increased mortality (Aro et al., 2011). Also isolated left anterior hemiblock is not a risk factor of cardiac morbidity or mortality (Elizari et al., 2007). The conduction delay of the His-Purkinje system can produce a small positive change to the end of the QRS complex or fragmentation of the S wave. ECG finding can be similar as the rSr′-morphology of Das et al. (2006). We suppose that an explanation to some of the fQRSs noticed on the grounds of the previous fQRS criteria results from the benign normal variants. We believe that fQRS occurring especially in the anterior precordial leads, as an rSr-morphology could be a benign finding. The same would be with the end-notched S waves in the lateral leads.

The separation of malignant phenotypes of fQRS from those which are thought to be benign would be a vital development step in prognostics of fQRS. Thus, it is an essential aim of our proposal and future studies. In a large cohort study discussing the silent MI (Zhang et al., 2016), it was shown that almost half of the subjects who developed a Q wave in their ECG during the follow-up were asymptomatic. Thus, a substantial part of myocardial infarctions are silent but the identification of the subjects with silent myocardial infarctions would be of high importance to initiate an effective secondary prevention. In the future, fQRS may serve as an additional marker of these infarctions. What is more, in cases of non-Q wave MI and old MIs the identifying of a myocardial scar and depolarization abnormalities should be based to fragmented QRS complex (Das et al., 2006; Michael et al., 2007; Fares et al., 2013). The detection of fQRS from the ECG of these patients would possible help to save the lives, at least to arrange the secondary prevention to these people. Often a myocardial scar and fibrosis are enclosed to participation of slower conduction areas (Mann et al., 2015). This is a prerequisite for the formation and sustaining of the ventricular tachycardia. Therefore, fQRS affords clinicians an important tool for the risk evaluation of SCD considering the fact that fQRS have proved to be linked to the risk of SCD in patients with the ischemic cardiomyopathy and HCM (Brenyo et al., 2012; Bonakdar et al., 2016).

For now, researchers have been able to prove, with a varying success, fQRS as a statistically significant ECG-marker for increased risk of mortality only when occurring in lateral leads of the subject with a CAD (Terho et al., 2014). We suppose that with the modified fQRS criteria we are able to maintain more precise information of prevalence and prognostics of the occurrence of the different fQRS phenotypes in the specific major coronary artery territory. Our hypothesis is that especially fQRSs in the inferior and lateral territories could predict the increased risk of the SCD. We suppose that fragmentation occurring in the R wave (notched R and RSR′) could be more significant finding than other fQRS morphologies. However, we do not want to exclude the possible importance of the notched S and R-S-borderline-fQRS, especially in anterior precordial leads V1 and V2. Our hypothesis is that all these fQRS morphologies (notched R, RSR′, notched S and R-S-borderline fQRS) could result from the myocardial scar which causes an intramyocardial conduction delay. Studying fragmentations in the Q wave could provide interesting aspects considering it has not been reported previously in the literature.

In short, we aim at identification of benign fQRS phenotypes from malignant phenotypes to improve accuracy of the prevalence and prognostic values of the fQRS. Later on we will enhance the modified fQRS criteria possible with a proper amplitude limits, occurrence of Q wave and demands of the QRS-axis. When all the characteristics of fQRS have been separated and validated also from other research materials than ours, the clear vision of benign phenotypes of fQRS can be shaped. After that it is possible to formulate simplified fQRS criteria for clinical use.

## Methods

By modified fQRS criteria described above our goal is to study the prognostic value of the fQRS in different populations. By using the both the fQRS criteria proposed by Das et al. (2006) and our novel modified fQRS criteria we will be able to identify potentially malignant fQRS patterns. The research material of our study is extensive. We will use the data of Mini-Finland Health Survey (*n* = 7217) collected by the Finnish Social Insurance Institution (National Institute for Health and Welfare, 2016). The initial examinations were carried out 1977–1980 in 40 different regions around the Finland and the subjects were over 30-year-old Finnish people. The baseline information has been collected by interviews and physical examinations and also standard 12-lead ECGs were recorded at paper speed 50 mm/s and calibration 10 mm/mV. The follow-up data of this population is available, the end-points will be all-cause mortality, cardiac mortality, and sudden cardiac death. (National Institute for Health and Welfare, 2016). All the measurements will be done by EASE ECG-analysis computer program developed to the need in question. The analysis made by EASE is based on visual definition of fragmentation amplitude and morphology. All the data will be analyzed by Statistical Package for Social Studies 21.0 program (SPSS).

In addition, research permission to another research material from the Institute of Health and Welfare, Health 2000 data (National Institute for Health and Welfare, 2009), is currently admitted. Health 2000 data will increase the coverage of our random general population. This study is very similar to Mini-Finland Health Survey. The initial examinations were carried out 2000–2001 and the material contains over 7217 standard ECGs from over 30-year-old Finnish subjects. The baseline information has been collected by interviews, health information comes from the updated registers and ECGs are recorded in the physical examinations. ECGs are similar compared to Mini-Finland data. The endpoint of the follow-up will be the same as above. (National Public Health Institute, 2000).

In addition, we are going to extend our research material to ARTEMIS and EU-CERT-ICD data. ARTEMIS data contains 1950 CAD patients with type 2 diabetes (National Institutes of Health, 2016). This case-control study data is collected during 2007–2014 in 2 and 5 year follow-ups and will help us to estimate the prognostic values of the fQRS in patients with CAD. In EU-CERT-ICD data all the subjects fulfill the criteria for the ICD placement and ICD have also been/will be implanted to some of the subjects. This data contains both a retrospective database (*n* = 2041) and the prospective material (EU-CERT-ICD, 2016). All the ECGs have been/will be recorded before ICD-implantation. The size of the prospective material is estimated to be approximately 2250 ECGs. We plan to compare the prevalence and prognostic values of fQRS in ARTEMIS and EU-CERT-ICD data (total *n* = 6241) to those in Mini-Finland and Health 2000 data (total *n* = 15,245). Later on the new fQRS criteria should also be validated by comparing the QRS fragmentation with cardiac imaging, e.g., magnetic resonance imaging with gadolinium late enhancement.

## Author contributions

MH wrote the article and developed the modified fQRS-criteria. AE participated in drafting the article and revising it critically and is also made substantial contributions to acquisition of data and analysis and interpretation of data. TK has developed the ECG analysis computer programme used analyzing the data and is also participated revising article. HH has, as the group leader, helped in drafting the article, and revising it critically.

## Funding

Funded in part by the grant no 602299 of FP 7 Health Program of EC (EU-CERT-ICD).

### Conflict of interest statement

The authors declare that the research was conducted in the absence of any commercial or financial relationships that could be construed as a potential conflict of interest.

## References

[B1] Adar A. Canyılmaz E. Kiris A. Ilter A. Serdar L. Memis Y.. (2015). Radiotherapy induces development of fragmented QRS in patients with breast cancer. Breast Care 10, 4. 10.1159/000431030 26600765 PMC4608666

[B2] Ahn M. S. Kim J. B. Yoo B. S. Lee J. W. Lee J. H. Youn Y. J.. (2013). Fragmented QRS complexes are not hallmarks of myocardial injury as detected by cardiac magnetic resonance imaging in patients with acute myocardial infarction. Int. J. Cardiol. 168, 3. 10.1016/j.ijcard.2012.12.086 23336958

[B3] Aro A. L. Anttonen O. Tikkanen J. T. Junttila M. J. Kerola T. Rissanen H. A.. (2011). Intraventricular conduction delay in a standard 12-lead electrocardiogram as a predictor of mortality in the general population. Circ. Arrhythm. Electrophysiol. 4, 5. 10.1161/CIRCEP.111.963561 21841194

[B4] Boineau J. P. Cox J. L. (1973). Slow ventricular activation in acute myocardial infarction. A source of re-entrant premature ventricular contractions. Circulation 48, 702–713. 10.1161/01.CIR.48.4.702 4126756

[B5] Bonakdar H. Moladoust H. Kheirkhah J. Abbaspour E. Assadian Rad M.. (2016). Significance of a fragmented QRS complex in patients with chronic total occlusion of coronary artery without prior myocardial infarction. Anatol. J. Cardiol. 16, 2. 10.5152/akd.2015.5887 26467369 PMC5336723

[B6] Brenyo A. Pietrasik G. Barsheshet A. Huang D. T. Polonsky B. McNitt S.. (2012). QRS fragmentation and the risk of sudden cardiac death in MADIT II. J Cardiovasc. Electrophysiol. 23, 12. 10.1111/j.1540-8167.2012.02390.x 22805297

[B7] Bulut M. Deniz Acar R. Ergün S. Geçmen Ç. Akçakoyun M. (2015). Cardiac rehabilitation improves the QRS fragmentation in patients with ST elevation myocardial infarction. J. Cardiovasc. Thorac. Res. 7, 3. 10.15171/jcvtr.2015.21 26430496 PMC4586605

[B8] Celikyurt U. Karauzum K. Sahin T. Agacdiken A. Vural A. Ural D. (2015). Association between resolution of fragmented QRS and response to cardiac resynchronization therapy. Ann. Noninvasive Electrocardiol. 20, 2. 10.1111/anec.12186 25039278 PMC6931892

[B9] Das M. K. El Masry H. (2010). Fragmented, Q. R. S., and other depolarization abnormalities as a predictor of mortality and sudden cardiac death. Curr. Opin. Cardiol. 25, 1 10.1097/HCO.0b013e328333d35d 19881337

[B10] Das M. K. Khan B. Jacob S. Kumar A. Mahenthiran J. (2006). Significance of a fragmented Q8RS complex versus a Q wave in patients with coronary artery disease. Circulation 113, 21. 10.1161/CIRCULATIONAHA.105.595892 16717150

[B11] Das M. K. Maskoun W. Shen C. Michael M. A. Suradi H. Desai M.. (2010). Fragmented QRS on twelve-lead electrocardiogram predicts arrhythmic events in patients with ischemic and nonischemic cardiomyopathy. Heart Rhythm 7, 1. 10.1016/j.hrthm.2009.09.065 20129288

[B12] Das M. K. Michael M. A. Suradi H. Peng J. Sinha A. Shen C.. (2009). Usefulness of fragmented QRS on a 12-lead electrocardiogram in acute coronary syndrome for predicting mortality. Am. J. Cardiol. 104, 12. 10.1016/j.amjcard.2009.07.046 19962466

[B13] Das M. K. Saha C. El Masry H. Peng J. Dandamudi G. Mahenthiran J.. (2007). Fragmented QRS on a 12-lead ECG: a predictor of mortality and cardiac events in patients with coronary artery disease. Heart Rhythm 4, 1385–1392. 10.1016/j.hrthm.2007.06.024 17954396

[B14] Debonnaire P. Katsanos S. Joyce E. Van Den Brink O. V. W. Atsma D. E. Schalij M.e t al. (2015). QRS fragmentation and QTc duration relate to malignant ventricular tachyarrhythmias and sudden cardiac death in patients with hypertrophic cardiomyopathy. J. Cardiovasc. Electrophysiol. 26, 5. 10.1111/jce.12629 25648421

[B15] EU-CERT-ICD (2016). EU-CERT-ICD Website. Available online at: http://www.eu-cert-icd.eu/

[B16] Elizari M. V. Acunzo R. S. Ferreiro M. (2007). Hemiblocks revisited. Circulation 115, 9. 10.1161/CIRCULATIONAHA.106.637389 17339573

[B17] Fares H. Heist K. Lavie C. J. Kumbala D. Ventura H. Meadows R.. (2013). Fragmented QRS Complexes—a novel but underutilized electrocardiograhic marker of Heart Disease. Crit. Pathw. Cardiol. 12, 4. 10.1097/HPC.0b013e31829e005d 24240545

[B18] Gong B. Li Z. (2016). Total mortality, major adverse cardiac events, and echocardiographic-derived cardiac parameters with fragmented QRS complex. Ann. Noninvasive Electrocardiol. 21, 4. 10.1111/anec.12325 26523941 PMC6931654

[B19] Güngör B. Özcan K. S. Karataş M. B. Şahin İ. Öztürk R. Bolca O. (2016). Prognostic value of QRS fragmentation in patients with acute myocardial infarction: a meta-analysis. Ann. Noninvasive Electrocardiol. 6, 604–612. 10.1111/anec.12357 27018003 PMC6931668

[B20] Homsi M. Alsayed L. Safadi B. Mahenthiran J. Das M. K. (2009). Fragmented QRS complexes on 12-lead ECG: a marker of cardiac sarcoidosis as detected by gadolinium cardiac magnetic resonance imaging. Ann. Noninvasive Electrocardiol. 14, 4. 10.1111/j.1542-474X.2009.00320.x 19804507 PMC6932232

[B21] Jain R. Singh R. Yamini S. Das M. K. (2014). Fragmented ECG as a risk marker in cardiovascular diseases. Curr. Cardiol. Rev. 10, 277–286. 10.2174/1573403X10666140514103451 24827794 PMC4040879

[B22] Konno T. Hayashi K. Fujino N. Oka R. Nomura A. Nagata Y.. (2015). Electrocardiographic QRS fragmentation as a marker for myocardial fibrosis in hypertrophic cardiomyopathy. J. Cardiovasc. Electrophysiol. 26, 10. 10.1111/jce.12742 26102305

[B23] Lorgis L. Cochet A. Chevallier O. Angue M. Gudjoncik A. Lalande A.. (2014). Relationship between fragmented QRS and no-reflow, infarct size, and peri-infarct zone assessed using cardiac magnetic resonance in patients with myocardial infarction. Can. J. Cardiol. 30, 2. 10.1016/j.cjca.2013.11.026 24461922

[B24] Ma X. Duan W. Poudel P. Ma J. Sharma D. X. Y. (2016). Fragmented QRS complexes have predictive value of imperfect ST-segment resolution in patients with STEMI after primary percutaneous coronary intervention. Am. J. Emerg. Med. 34, 3. 10.1016/j.ajem.2015.11.010 26643157

[B25] MacAlpin R. N. (2010). The fragmented QRS: does it really indicate a ventricular abnormality? J. Cardiovasc. Med. 11, 11. 10.2459/JCM.0b013e32833b9816 20543708

[B26] Macfarlane P. W. Antzelevitch C. Haissaguerre M. Huikuri H. V. Potse M. Rosso R.. (2015). The early repolarization pattern: a consensus paper. J. Am. Coll. Cardiol. 66, 4. 10.1016/j.jacc.2015.05.033 26205599

[B27] Maheshwari S. Acharyya A. Puddu P. E. Mazomenos E. B. Leekha G. Maharatna K.. (2013). An automated algorithm for online detection of fragmented QRS and identification of its various morphologies. J. R. Soc. Interface 10, 89. 10.1098/rsif.2013.0761 24132202 PMC3808554

[B28] Malik M. (2013). Electrocardiographic smoke signals of fragmented QRS complex. J. Cardiovasc. Electrophysiol. 24, 11. 10.1111/jce.12226 24016353

[B29] Mann D. L. Zipes D. P. Libby P. and O. Bonow R. (2015). Arrhythmias, sudden death, and syncope, in Braunwald's Heart Disease: A Textbook of Cardiovascular Medicine, 10th Edn., ed Mann D. L. (Philadelphia, PA: Saunders), 5.

[B30] Michael M. A. El Masry H. Khan B. R. Das M. K. (2007). Electrocardiographic signs of remote myocardial infarction. Prog. Cardiovasc. Dis. 50, 198–208. 10.1016/j.pcad.2007.05.003 17976504

[B31] Morita H. Kusano K. F. Miura D. Nagase S. Nakamura K. Morita S. T.. (2008). Fragmented QRS as a marker of conduction abnormality and a predictor of prognosis of Brugada syndrome. Circulation 118, 17. 10.1161/CIRCULATIONAHA.108.770917 18838563

[B32] National Institutes of Health, P (2016). Prognosis of Type 2 Diabetic Patients (ARTEMIS). Available online at: https://clinicaltrials.gov/ct2/show/NCT01426685?term=NCT01426685&rank=1

[B33] National Public Health Institute, P (2000). The Report of the Methods. The execution, data and methods of the Health 2000 study. Available online at: http://www.julkari.fi/bitstream/handle/10024/78181/2005b6.pdf?sequence,=1

[B34] National Institute for Health Welfare (2009). Health 2000. Available online at: http://www.terveys2000.fi/indexe.html

[B35] National Institute for Health Welfare (2016). Mini-Finland Health Survey. Available online at: https://www.thl.fi/en/web/thlfi-en/research-and-expertwork/population-studies/finnish-mobile-clinic/mini-finland-health-survey

[B36] Sha J. Zhang S. Tang M. Chen K. Zhao X. Wang F. (2011). Fragmented QRS is associated with all-cause mortality and ventricular arrhythmias in patient with idiopathic dilated cardiomyopathy. Ann. Noninvasive Electrocardiol. 16, 3. 10.1111/j.1542-474X.2011.00442.x 21762255 PMC6932517

[B37] Terho H. K. Tikkanen J. T. Junttila J. M. Anttonen O. Kenttä T. V. Aro A. L.. (2014). Prevalence and prognostic significance of fragmented QRS complex in middle-aged subjects with and without clinical or electrocardiographic evidence of cardiac disease. Am. J. Cardiol. 114, 1. 10.1016/j.amjcard.2014.03.066 24819902

[B38] Torigoe K. Tamura A. Kawano Y. Shinozaki K. Kotoku M. Kadota J. (2012). The number of leads with fragmented QRS is independently associated with cardiac death or hospitalization for heart failure in patients with prior myocardial infarction. J. Cardiol. 59, 1. 10.1016/j.jjcc.2011.09.003 22019275

[B39] Wang D. D. Buerkel D. M. Corbett J. R. Gurm H. S. (2010). Fragmented QRS complex has poor sensitivity in detecting myocardial scar. Ann. Noninvasive Electrocardiol. 15, 4. 10.1111/j.1542-474X.2010.00385.x 20946552 PMC6931930

[B40] Wang D. D. Tibrewala A. Nguygen P. Swadia T. Jacobsen G. Khan A.. (2014). Fragmented QRS on surface electrocardiogram is not a reliable predictor of myocardial scar, angiographic coronary disease or long term adverse outcomes. Cardiovasc. Diagn. Ther. 4, 4. 10.3978/j.issn.2223-3652.2014.08.03 25276613 PMC4147086

[B41] Yan G. H. Wang M. Yiu K. H. Lau C. P. Zhi G. Lee S. W.. (2012). Subclinical left ventricular dysfunction revealed by circumferential 2D strain imaging in patients with coronary artery disease and fragmented QRS complex. Heart Rhythm 9, 6. 10.1016/j.hrthm.2012.01.007 22245798

[B42] Zhang Z.-M. Rautaharju P. M. Prineas R. J. Rodriguez C. J. Loehr L. Rosamond W. D.. (2016). Race and sex differences in the incidence and prognostic significance of silent myocardial infarction in the Atherosclerosis Risk in Communities (ARIC) Study. Circulation 133, 22. 10.1161/CIRCULATIONAHA.115.021177 27185168 PMC4889519

[B43] Zipes D. P. Das M. K. (2009). Fragmented QRS: a predictor of mortality and sudden cardiac death. Heart Rhythm 6, 3. 10.1016/j.hrthm.2008.10.019 19251229

